# Viral cultures for assessing airborne infectiousness of SARS-CoV-2: a systematic review and meta-analysis

**DOI:** 10.1186/s12879-025-12430-z

**Published:** 2025-12-25

**Authors:** Igho J. Onakpoya, Annette Plüddemann, Elena C. Rosca, Sara Gandini, Susanna Maltoni, Jon Brassey, Tom Jefferson, Carl J. Heneghan, David H. Evans, John M. Conly

**Affiliations:** 1 Department for Continuing Education, University of Oxford, Oxford, UK; 2 Centre for Evidence Based Medicine, Nuffield Department of Primary Care Health Sciences, University of Oxford, Oxford, UK; 3 Victor Babes University of Medicine and Pharmacy, Timisoara, Romania; 4 Department of Experimental Oncology, IEO European Institute of Oncology IRCCS, Milan, 20141 Italy; 5 Clinical Trial Centre Unit, IRCCS Azienda Ospedaliero - Universitaria di Bologna, Bologna, Italy; 6 Trip Database Ltd, Newport, UK; 7 Department of Medical Microbiology & Immunology, Li Ka Shing Institute of Virology, University of Alberta, Edmonton, Alberta T6G 2E1 Canada; 8 Departments of Medicine, Microbiology, Immunology & Infectious Diseases, and Pathology & Laboratory Medicine, Snyder Institute for Chronic Diseases and O’Brien Institute for Public Health, Cumming School of Medicine, University of Calgary and Alberta Health Services, Calgary, Canada

**Keywords:** SARS-CoV-2, COVID-19, Airborne, Transmission, Viral culture, Systematic review

## Abstract

**Introduction:**

There is uncertainty about the quantification, viability and infectivity of SARS-CoV-2 in air samples. Our objective was to systematically review the evidence for air sample virus infectiousness with high-level confirmatory studies.

**Methods:**

We conducted literature searches in LitCovid, medRxiv, PubMed, the WHO Covid-19 databases, and Google Scholar. We included studies that assessed viral infectiousness in the air using viral culture or serial qRT-PCR with or without genomic sequencing. Our primary outcome was the proportion of culture-positive air samples of SARS-CoV-2. Secondary outcomes explored the relationship between infectiousness and Cycle threshold (Ct). We used published methods for assessing quality, and R software for meta-analysis.

**Results:**

We included 26 studies that used viral culture to assess air sample positivity of SARS-CoV-2. The overall reporting quality was moderate. The overall pooled frequency of positive viral cultures was 14% (95% CI 7–17, I^2^ = 52.3%; *p* = 0.001). The data were not sufficient to compute a threshold for infectivity, or to explore the relationship between distance and infectiousness.

**Conclusions:**

The proportion of positive SARS-CoV-2 viral cultures following positive RNA samples in the air is low, suggesting that while viral RNA may be present, the likelihood of detecting culturable, infectious viruses is substantially lower. Our findings underscore the need for standardized guidelines to assess and report the infectivity and potential for transmissibility of airborne viruses, including the consistent reporting of Ct values and methods to mitigate bias.

**Supplementary information:**

The online version contains supplementary material available at 10.1186/s12879-025-12430-z.

## Introduction

Airborne transmission of an infectious agent is caused by the dissemination of droplet nuclei (aerosols) that contain microorganisms that remain infectious when suspended in the air over long distances and time. Given its potential for transmitting SARS-CoV-2, we previously published a systematic review to identify, appraise and summarise the evidence on airborne transmission [1]. This review included 67 primary studies and 22 reviews: all included studies were observational, of very low to low quality, and lacked standardised methods. While ten studies attempted viral culture, the general lack of recoverable SARS-CoV-2 virus in cultured samples prevented us from drawing any firm conclusions, highlighting a critical evidence gap, to support airborne transmission similar to other studies [2, 3]. Most primary studies in these reviews detected SARS-CoV-2 RNA in the air, but not infectious virus, and the presence of RNA does not infer infectiousness.

A systematic review from our group assessed potential transmission from asymptomatic individuals by including information on serial PCR cycle threshold (Ct) readings and/or viral culture and/or gene sequencing [4]. However, the available evidence was limited regarding the infectious dose and relative frequency of the transmission events.

Since the publication of our first review on airborne transmission [1], several new studies that include infectious SARS-CoV-2 status have been published. We therefore conducted this systematic review to examine the potential for airborne transmission with high-level confirmatory studies of infection that include viral culture and/or longitudinal serial PCRs with or without gene sequencing. To assess the potential of airborne transmission of SARS-CoV-2, we aimed to address the following questions:

1. Are airborne samples infectious?
2. If so, what proportion are infectious, and what is the distance and duration of infectiousness in air samples?
3. What is the relationship between infectiousness and PCR cycle threshold (Ct)?
4. Is there evidence of a chain of transmission that establishes an actual instance of airborne transmission of SARS-CoV-2?
5. What circumstances might facilitate infectious viruses being airborne over long distances?

## Methods

The protocol for this systematic review has previously been published at https://www.medrxiv.org/content/10.1101/2022.01.28.22270021v1.

We conducted searches in four databases: LitCovid, medRxiv, Google Scholar, and the WHO Covid-19 database, using the terms aerosol OR airborne OR inhalation OR air OR droplet and viral replication, viral culture, viral transmission and various synonyms to 31 December 2024 (see Appendix 1). An information specialist [JB] conducted the searches. We undertook forward citation to identify relevant articles. We included primary studies reporting “airborne transmission” by attempting viral culture or serial qRT-PCR with or without genomic sequencing. We excluded predictive or modelling studies. Our primary outcome was the proportion of SARS-CoV-2 viral cultures in air samples. Our secondary outcomes were to investigate the relationship between viral culture and cycle thresholds (Ct), and to determine if there was evidence of an actual chain of transmission of SARS-CoV-2, as well to identify any specific factors that could facilitate the carriage of replication-competent SARS-CoV-2 over long distances.

Three reviewers (CJH, IJO, JB) independently screened study abstracts to determine eligibility. Any disagreements were resolved through discussion.

### Data extraction and quality assessment

We extracted variables using a customised data extraction spreadsheet in Excel. We extracted information on the study characteristics, population, settings, the environment in which the cultures were obtained, methods, and the main results from included studies. We also extracted data on the sampling methods including quantitative source-to-sampling distance measures, RT-PCR samples for SARS-CoV-2 RNA including Ct and copies per m^3^ or other quantitative measures of sampled air, viral culture methods and results confirming the virus as SARS-CoV-2, size of particles (when reported) and proportion of positive RT-PCR tests in the samples. One reviewer (IJO) extracted the data, with independent verification by a second reviewer (CJH).

To judge the quality of included studies, we assessed the following domains: (i) Source population – was the population recruited into the study clearly described? (ii) Methods – did the study authors sufficiently describe the methods used to enable replication of the study? (iii) Sample sources – were the sources for the air samples clear? (iv) Outcome reporting – was the analysis of the results appropriate, and (v) Follow-up – was the pattern and number of air samples sufficient to demonstrate airborne infectiousness and a chain of transmission from human-to-human?

One reviewer (IJO) assessed study quality while a second reviewer (CJH) independently verified the assessments. Disagreements were resolved via consensus.

To assess the chain of transmission (question 4), we used methods based on our previously published reviews on causality assessments [4, 5] and information from previously published reviews on adequate follow-up and reporting of symptoms and signs of COVID-19 infection [6, 7].

### Data synthesis

We displayed the quality assessment using bar charts and computed the proportion of positive and negative viral culture results using frequencies. Using R software (inverse variance method), we calculated the mean frequency of positive viral culture tests, presenting the results using both fixed and random effects estimates. We used continuity correction of 0.5 in studies with zero cell frequencies. We assessed heterogeneity using the I-square statistic: values of 25%, 50% and 75% which corresponded to mild, moderate and significant heterogeneity, respectively. We used trim and fill analysis by study weight to explore heterogeneity and test the robustness of overall estimates, and funnel plot to test for publication bias.

## Results

**Fig. 1:**
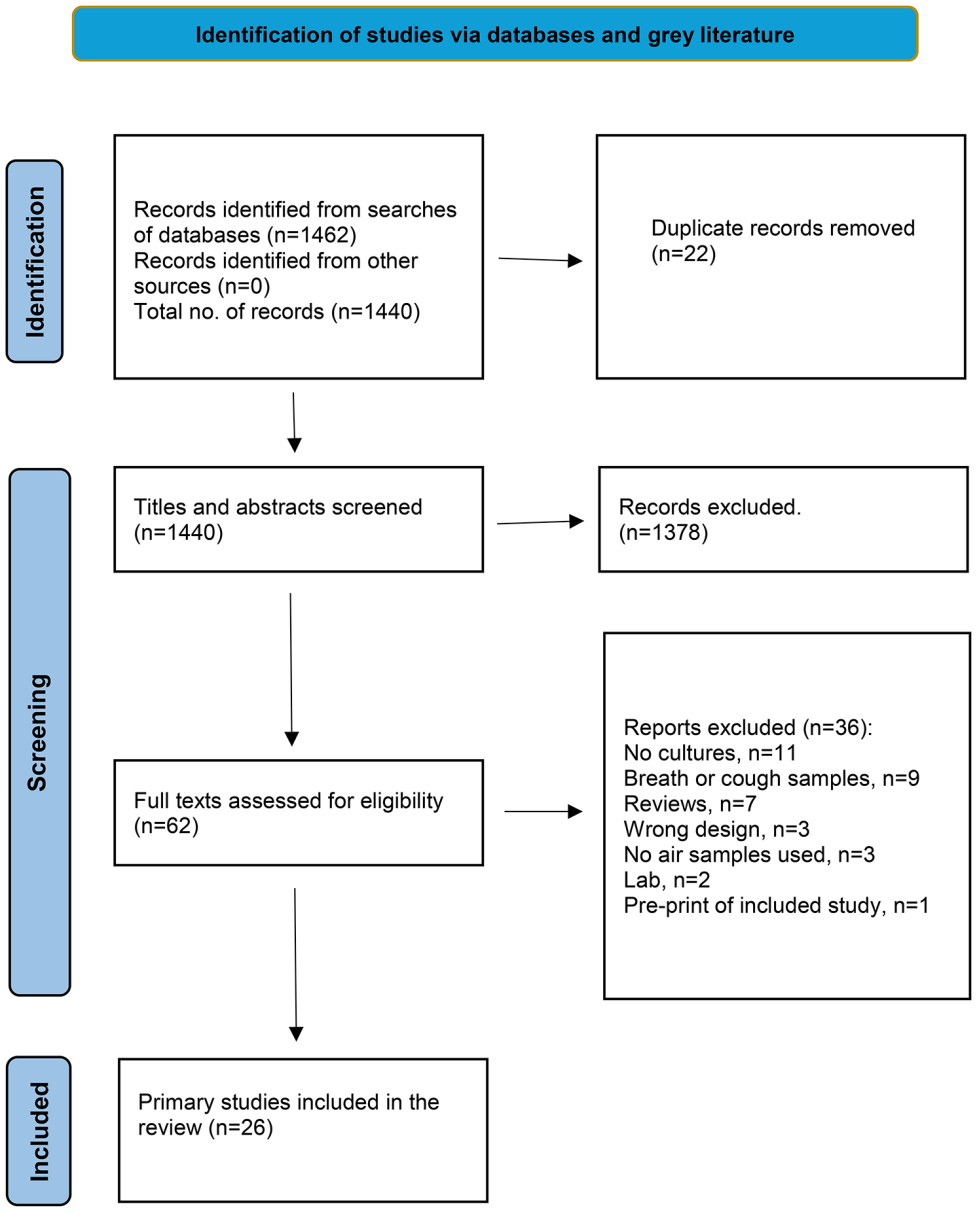
PRISMA 2020 flow diagram for inclusion of studies investigating airborne transmission of SARS-CoV-2 through viral cultures. *From:* Page MJ, McKenzie JE, Bossuyt PM, Boutron I, Hoffmann TC, Mulrow CD, et al. The PRISMA 2020 statement: an updated guideline for reporting systematic reviews. BMJ 2021;372:n71. 10.1136/bmj.N71. For more information, visit: http://www.prisma-statement.org/

Our electronic searches identified 1440 non-duplicate citations, of which 62 eligible studies were identified (Fig. 1). We excluded 11 studies for not performing viral culture, nine for using cough and breath samples, and seven for being reviews. Three studies were excluded due to inappropriate design, three for not culturing air samples, two for being laboratory studies exploring mechanistic transmission, and one for being a preprint of a previously included study (see Appendix 2 for a complete list of excluded studies). Finally, the review included 26 studies [8–33].

Table 1 reveals the key details of the included studies, all of which were observational in design. Eighteen studies were conducted exclusively in healthcare settings, and three exclusively in residential settings. Other settings included a nursing home (*n* = 1), a testing room (*n* = 1), a car (*n* = 1), a hospital, and a nursing home (*n* = 1), as well as a hospital and a community isolation room (*n* = 1). In one study (Fortin 2023), the samples were stored for 14 months before culture. The number of specimens ranged from 2 to 360 across the studies, and the frequency of positive RNA air samples varied from 2% to 100%.

**Table 1:** Characteristics of studies assessing the transmission of SARS-CoV-2 through viral cultures

Study ID	Setting	Source population & specimen source	Total number of sampled specimens	Culture methods	Total number of specimens cultured
Abubakar-Waziri H 2023	Hospital	ICUs with adults suffering from COVID-19, respiratory wards, and public waiting areas.	207	Vero E6 grown in Dulbecco’s Modified Eagle’s Medium	20
Ang AX 2021	Hospital	Naturally ventilated open cohort wards (2) and mechanically ventilated isolation ward (1)	27	Vero E6 grown in Dulbecco’s Modified Eagle’s Medium	27
de Sousa NR 2022	Hospital	Infectious disease ward	44	Vero E6 grown in Dulbecco’s Modified Eagle’s Medium	25
Fortin A 2023	Hospital	Hospital rooms	30	VERO E6 cells were inoculated with 150 PFU	4
Gohli J 2022	Testing room	Testing room	14	Vero E6 grown in Dulbecco’s Modified Eagle’s Medium	7
Gohli J 2022a	University Hospital	Ward at the Department of Infectious Diseases Hospital	13	Vero E6 grown in Dulbecco’s Modified Eagle’s Medium	2
Kitagawa H 2022	Hospital	Hospital rooms of patients with COVID-19 with an early infection	18	Vero E6/TMRPSS2 cells	18
Kotwa JD 2021	Hospital	Rooms of 78 inpatients with COVID-19 at 6 acute care hospitals	146	Vero E6 grown in Dulbecco’s Modified Eagle’s Medium	3
Kuloğlu ZE 2023	Hospital	Indoor air samples at University Hospital	33	Vero E6 grown in Dulbecco’s Modified Eagle’s Medium	10
Lebreil AL 2021	Hospital	Single-bed ICU rooms of adult patients with COVID-19	40	Vero E6 grown in Dulbecco’s Modified Eagle’s Medium	12
Lednicky JA 2021a	Clinic in University Health Centre	Respiratory infection evaluation area of University Health Centre	2	Vero E6 grown in Dulbecco’s Modified Eagle’s Medium	2
Lednicky JA 2021b	Car	Air within a car driven by a patient infected with SARS-CoV-2	5	Confluent Vero E6 cells	4
Linde KJ 2022	Nursing home	Air in rooms of COVID-19 infected nursing home residents	360	Vero cells, clone 118 - virus culture medium was added	41
Mallach G 2021	Hospitals and long-term care homes	Hospital rooms, exhaust (return) air ducts drawing air from inside penitentiary living areas, and long-term care home resident rooms	99	Vero E6 grown in Dulbecco’s Modified Eagle’s Medium	15
Moharir SC 2022	Hospital	Hospitals and community with COVID-19 patients	160	Vero E6 grown in Dulbecco’s Modified Eagle’s Medium	3
Nagle S 2022	Hospital	Hospital rooms of patients with acute COVID-19	59	Vero cells	7
Ong SW 2021	Hospital rooms and community isolation facilities	Hospital rooms and community isolation facilities	12	Vero C1008 cells in Eagle’s MEM	6
Otter JA 2022	Acute healthcare setting	ED, ICU, acute admissions unit, ward, entrance and public area	27	Vero E6 grown in Dulbecco’s Modified Eagle’s Medium	1
Santarpia JL 2021	Hospital	Hospital rooms of COVID-19 patients	18	Vero E6 grown in Dulbecco’s Modified Eagle’s Medium	18
Shankar SN 2022	Residential rooms of self-isolating persons	Residential rooms of two volunteers with COVID-19	20	Vero cells	6
Tan KS 2023	Hospital	Hospital environment	28	12-well plate of confluent A549–ACE2	3
Vass WB 2022	Residential room of self-isolating person	Residential setting with a self-isolating college student with COVID-19	9	VeroE6 cells in aDMEM (advanced Dulbecco’s modified essential medium	5
Vass WB 2023	Residential settings of individuals with COVID-19	Residences occupied by individuals with COVID-19	43	Vero E6 cells and LLC-MK2 cells, plaque assay	43
Winslow RL 2021	Hospital	30 hospitalised patients with COVID-19 requiring supplemental oxygen	90	Vero E6 grown in Dulbecco’s Modified Eagle’s Medium	4
Zhou J 2022	Acute healthcare setting	Hospital	31	Vero E6 grown in Dulbecco’s Modified Eagle’s Medium	14
Zhou J 2023	Hospital	Air samples of patients with COVID-19 at quarantine unit	252	Vero E6 grown in Dulbecco’s Modified Eagle’s Medium	63

In 17 studies (65.4%), the severity of symptoms varied from no symptoms (1 study) to severe symptoms (see Appendix Table 1). In three studies, the setting in which air sampling occurred involved patients with severe COVID-19 infection. The patients in one study [13] were all on nebulizer therapy [13], another involved patients with COVID-19-induced acute respiratory distress syndrome (ARDS) [17], and a third included patients requiring supplemental oxygen [31]. Up to 15 different types of air samplers were employed across the studies (see Appendix Table 1). The study duration ranged from 15 minutes to eight months (≥2 intervals). Nine studies performed sampling across two or more seasons. Twenty-one studies (80.8%) reported data on sampling distances ranging from 0.5 meters (m) to > 14 m across the studies; only 10 studies reported specific sampling distances.

Thirteen studies (50%) reported Ct values corresponding to culture results; however, the data were insufficient for conducting a meta-analysis, primarily because studies either reported Ct values for only positive or only negative cultures (but not both) or they did not report specific Ct cut-off values. Five studies reported the viral concentrations (viral RNA) of positive culture samples, while six reported the concentrations of negative culture samples. There were variations in the metrics used to present viral concentrations in the culture samples across the studies (see Table 1). The viral concentration was either not reported or unclear in 15 studies (57.7%). Twenty-five studies employed VERO E6 cells for viral culture.

**Fig. 2:**
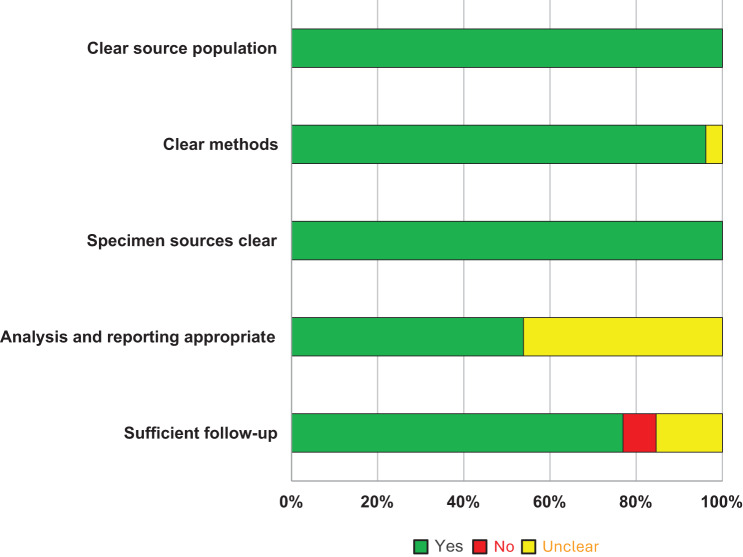
Reporting quality of studies investigating airborne transmission of SARS-CoV-2 through viral cultures

Twenty-five studies adequately reported the methods used for sample collection, and all studies clearly described source populations and the sites of air samples. Fourteen studies reported results and analysed the data in sufficient detail, and 20 (77%) demonstrated adequate follow-up. Overall, the reporting quality of the included studies was moderate (see Fig. 2).

**Fig. 3:**
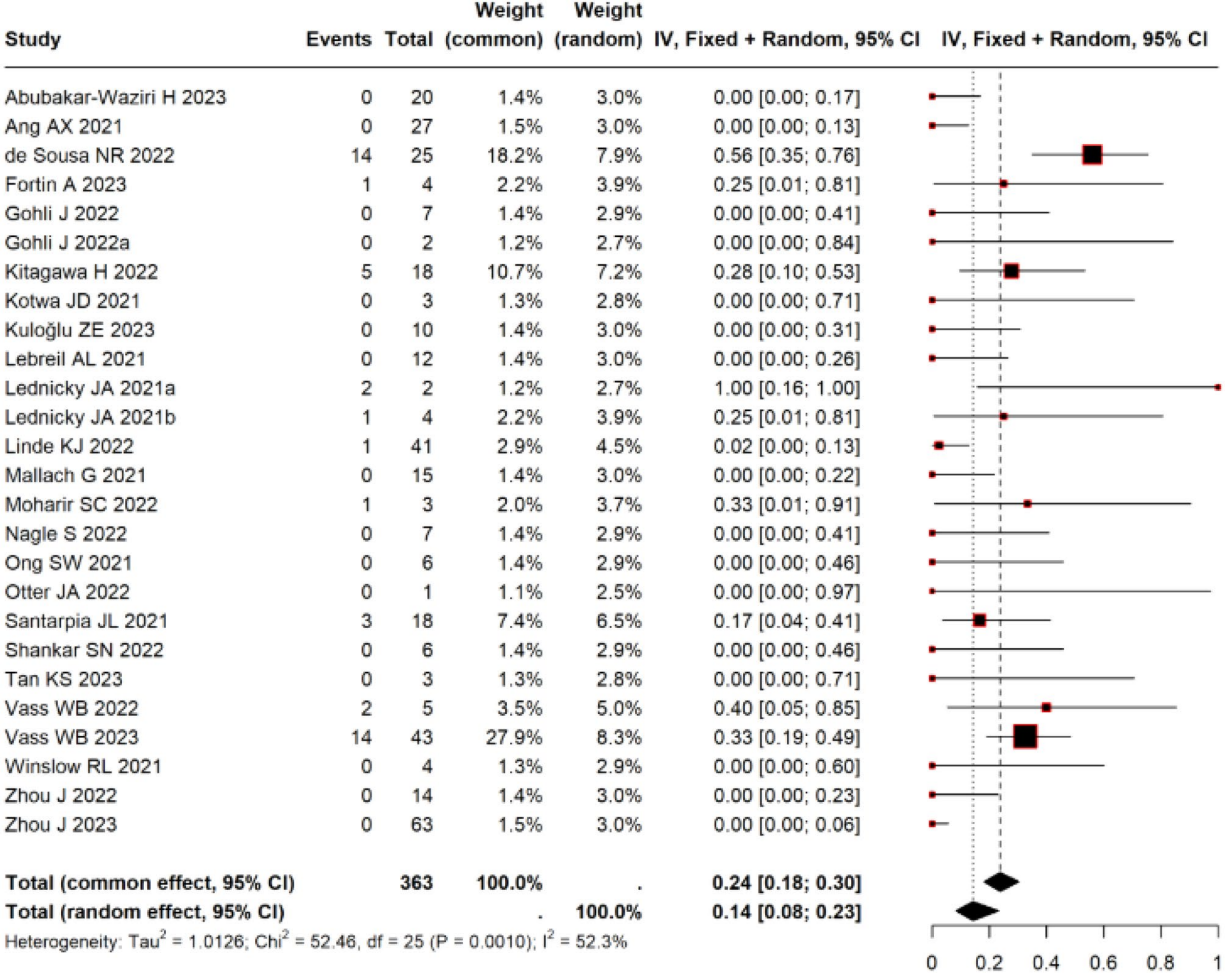
Forest plot showing the frequency of positive SARS-CoV-2 viral culture results following positive PCR

The frequency of viral cultures ranged from 0% to 100% for either positive or negative viral cultures. The majority (61.5%) of the studies did not detect positive viral cultures in samples. The overall frequency of positive viral cultures was 14% (95% CI 7 to 17%, I^2^ = 52.3%; Fig. 3). The removal of three studies that carried the largest weights [10, 14, 30] resulted in similar results, but with a marked reduction in heterogeneity (I^2^ = 14%; appendix Fig. 1). A funnel plot (Appendix Fig. 2) showed symmetrical distribution of studies around the pooled effect estimate but does not rule out the risk of publication bias. No study used serial Cts based on RT-PCR to confirm the results of positive viral cultures. In one study [14], the RNA concentration was significantly higher in samples in which viable SARS-CoV-2 was detected compared to samples without viable virus (median RNA concentration 5.5 × 10^5^ copies/m^3^ vs. 2.4 × 10^2^ copies/m^3^, respectively *p* = 0.027; see Appendix Table 1). Another study [16] reported a significant difference in mean viral loads in rooms of patients wearing masks vs. no masks: 1.45 × 10^2^ PFU *m*–3 vs. 3.60 × 10^10^ PFU *m*–3; the method used to compute the viral load from Ct values was not described in sufficient detail to permit a re-examination of these numbers.

Eight studies (30.8%) used genome sequencing to determine the strain of cultured viruses (see Appendix Table 1). Five of the eight studies showed positive results that were consistent with the strains from viral culture. One study reported positive sequencing results despite negative viral culture, while another reported negative sequencing results despite positive viral cultures.

## Discussion

We identified 26 studies that investigated airborne infectiousness of SARS-CoV-2 through viral culture. The overall frequency of positive SARS-CoV-2 viral cultures following positive RNA samples was 14%, suggesting that while airborne viral RNA is often detectable, infectious virus was found only in a small proportion of airborne samples. The reporting quality across the included studies was moderate. We were unable to assess the relationship between sampling distance and infectiousness because of insufficient data. We were also unable to explore the relationship between Ct and infectiousness because of the paucity of data. No study demonstrated conclusive evidence of a chain of transmission establishing an actual instance of airborne transmission of SARS-CoV-2 within the reported studies.

During the COVID-19 pandemic, there was considerable debate about the role played in transmission of SARS-CoV-2 via the airborne route, particularly over longer distances versus other routes of transmission. Our initial review had concluded that there was insufficient evidence to firmly determine the role of the airborne route in the transmission of SARS-CoV-2 and that further research was required using standardized guidelines for conducting and reporting research on airborne transmission [1]. Other authors also supported that the infectiousness of airborne SARS-CoV-2 was uncertain because of poor sampling methodology [34]. Most of the studies that reported the airborne route as the main route of transmission were based on hypotheses, case reports and opinions [35–37]. The results of our review indicate that while SARS-CoV-2 can retain infectiousness based on viral culture results of air samples in some settings, the frequency of transmission events based on the results of viral culture positivity in air is unknown as is the minimum infectious dose, the durability of infectious virus in the air, airflow dynamics and the effects of humidity, settling velocity, temperature and other environmental factors. Studies of the retention of infectious SARS-CoV-2 in air have demonstrated a very short duration of infectivity, with a substantive decrease in infectivity to ∼10% of the starting value observed over 20 min, with the majority of the loss occurring within the first 5 min after aerosolization [38] However, the minimum infectious dose of SARS-CoV-2 within a cubic metre of air has not been established.

Some authors have stated that the lack of culturability of SARS-CoV-2 does not imply non-infectiousness because of difficulty with culturing the virus [39]. Our results suggest that the capture of viral cultures from the air is a technical issue that can be overcome, countering these arguments. Furthermore, the use of an intranasal inoculum dose of 10 TCID50 (equivalent to ~ 7 PFU) used in the only human challenge study of SARS-CoV-2 to date, was shown to induce active infection in 53% of seronegative participants [40], providing evidence to support a minimal infectious dose of 5–10 PFU, consistent with human challenge studies of other humanCoVs [41]. These latter studies provide valuable information on what quantitative levels of infectious SARS-CoV-2 may be used in assessing the relevance of air samples and indicate that cell culture from air samples can be used for enumerating any infectious virus that may be present.

### Comparison with the existing literature

Our findings are consistent with those of other reviews, suggesting that there may be a risk of airborne transmission of SARS-CoV-2 in indoor environments [42–45] but specific details were often lacking. These latter systematic reviews varied considerably in their quality assessments and in their primary research questions. The review by Noorimotlagh et al. [42] primarily focused on experimental studies, with no quality filters applied to the primary studies. It acknowledged that all included primary studies had limitations and assumptions, and there was limited epidemiologic data. The study by Duval et al. [43] used a quality assessment with GRADE focusing on epidemiologic features suggesting airborne transmission but the majority of the studies were of very low to low certainty and in most of the studies, other routes of transmission could not be ruled out. The reviews by Francis et al. [44] and Madewell et al. [45] focused on epidemiologic observational studies that assessed secondary transmission rates and focused their quality assessment on that domain and did not comment on other potential confounding modes of transmission. The majority of the assessed studies were rated as low to moderate in quality.

In contrast to these reviews relying mainly on epidemiologic evidence of generally low quality, we used viral culture to assess the frequency of positive air samples. Our results are also consistent with the findings of another review, which showed that only 1/16 (6%) positive SARS-CoV-2 RNA air samples were positive for viral culture [46]. However, that review included only three studies, compared to the 26 in our review. We also examined whether a definitive chain of transmission could be found in any of the studies and found none.

Results from previous studies using ferrets [47] have demonstrated transmission of SARS-CoV-2 via the aerosol route through a connecting duct at a one-metre distance, but with the use of very high unidirectional airflow (100 l/min), which makes the findings difficult to interpret in more natural settings. Experimental studies using Syrian Golden hamsters demonstrated that SARS-CoV-2 transmitted efficiently between infected hamsters and non-infected hamsters by both direct contact and via aerosols [48]. When the hamsters were caged separately, 1.8 cm apart, but again with conditions of high airflow rates, the naive hamsters were found to be efficiently infected via close contact, presumably via aerosolized transmission. Bao and colleagues [49] using the human angiotensin-converting enzyme 2 (hACE2) transgenic mouse model (best representing the human setting by virtue of the human receptor) found direct contact and direct droplet deposition (co-housed infected and non-infected mice in the same cage or separate cages at close distances) was highly efficient for transmission of SARS-CoV-2. However, aerosolized transmission using a bioaerosol generator with a very high burden of virus at 1.4 x10^6^ pfu/ml was far less efficient and required 25 minutes of continuous exposure before infection could be seen in exposed mice. Similar findings have been reported by other authors [50, 51]. A more recent study [52] using a human-to-K18-hACE2 mouse model with the mice co-housed in close proximity in the hospital rooms of patients infected with SARS-CoV-2 with saliva/sputum samples at concentrations of 6.4x10^3^ to 6.4x10^5^ pfu/ml found no evidence of transmission to the mice after four continuous hours of exposure. These latter studies provide some evidence regarding the relative inefficiency of airborne transmission of SARS-CoV-2. The inability to explore the association between distance and infectiousness in our review, due to inconsistent reporting in the primary studies, makes any conclusion about the distance-transmission relationship in humans conjectural at best.

### Strengths and limitations

To our knowledge, this is the most comprehensive and up-to-date review that investigates the potential for airborne transmission of SARS-CoV-2 using viral culture and provides point estimates of the frequency of the presence of infectious SARS-CoV-2 in air samples. We conducted extensive searches to identify relevant studies, and we accounted for the quality of included studies. However, we recognize several limitations. We may not have identified all studies that assessed airborne transmission of SARS-CoV-2 through viral culture, especially unpublished studies. We were unable to explore the relationship between sampling distance and infectiousness because of inadequate data. We could not determine the threshold for infectiousness of airborne SARS-CoV-2 samples due to the paucity of data in the reviewed primary studies. The lack of adequate controls and disparities in correlation between Ct values and viral culture outcomes across the studies also clouds the issue. Furthermore, we could not assess for how long positive air culture samples remain infectious. Finally, the failure of the majority of studies to perform sequencing prevents us from drawing firm conclusions about transmission dynamics for positive viral culture results.

### Implications for research

#### Study designs

We observed variations in the sampling methodology across the included studies. Recent reviews of airborne virus sampling methods reinforce the need for methodological standardization in studies evaluating SARS-CoV-2 infectiousness. Systematic assessments by Silva et al. [53] and Dias et al. [54] demonstrate substantial heterogeneity in sampler types, sampling volumes, flow rates, transport conditions, and environmental reporting, which limits comparability across studies and complicates interpretation of negative culture results. Experimental work comparing sampling devices has shown that the collection of infectious virus is especially sensitive to the choice of sampler. Liquid-based collectors such as impingers and cyclone devices generally preserve viability more effectively than filters, which, although efficient at capturing particles, frequently lead to desiccation and structural degradation of enveloped viruses. Comparative studies using influenza viruses and surrogates (e.g., bacteriophage Phi6) consistently highlight the importance of assessing not only capture efficiency but also recovery efficiency, as recovery rates vary markedly across devices [55]). Foundational aerobiology research and more recent optimization studies also demonstrate that viral viability is strongly influenced by environmental factors, particularly relative humidity and temperature [38, 54, 56], yet these parameters were inconsistently reported or entirely absent in many primary SARS-CoV-2 studies included in our review. Taken together, these findings underscore the feasibility and necessity of a standardized approach incorporating (1) validated liquid-based samplers, (2) mandatory environmental logging, and (3) validation with surrogate viruses to ensure that the absence of culturable virus reflects true environmental conditions rather than methodological loss, and (4) quantitation of the amount of virus present in the samples.

Some authors have advocated the development of standardized protocols for air sampling of SARS-CoV-2 [53, 54, 57]. Human challenge studies examining airborne transmission of viruses over varying distances (including the use of human-to-human or human-to- K18-hACE2 mouse studies) should also be a priority for future research [33, 58].

#### Reporting standards

Future viral culture studies should report Ct values, along with the primers used and the assay parameters for both positive and negative culture results to help establish a reliable threshold for infectiousness and transmission. Ideally these Ct values should be reported as RNA quantities, using a conversion based on internal standards, to eliminate differences in the sensitivity of different PCR assays. More precise reporting of sampling distances is required for exploring and determining any relationships between distance and infectivity, which could be beneficial for infection control practices.

At present, there is no universally agreed set of guidelines for reporting studies of human transmission of airborne viruses. This lack of standardisation hampers the comparability and reproducibility of research in this field. Historically, efforts to address poor reporting practices in research have led to the independent development of reporting guidelines. For instance, in 1994, two separate trial reporting guidelines were introduced: the Standards of Reporting Trials (SORT) Statement and the Alisomar guidelines, developed by the Asilomar Working Group on Recommendations for Reporting of Clinical Trials in the Biomedical Literature [59]. Recognising the benefits of a unified approach, the groups responsible for these initiatives collaborated to produce the CONSORT (CONsolidated Standards Of Reporting Trials) Statement, which was first published in 1996, then revised in 2001 and again in 2010. Additionally, a CONSORT Explanation and Elaboration document was released to clarify and illustrate the guiding principles behind the statement. Similar collaborative efforts across different regions should be prioritised for developing standardised reporting guidelines for transmission studies. Such an approach would improve the quality, transparency, and comparability of research on airborne virus transmission.

### Implications for practice and policy

We found that only a small proportion of positive airborne samples of SARS-CoV-2 from viral culture are infectious. The threshold Ct value at which SARS-CoV-2 exists within air samples of SARS-CoV-2 to become infectious is unknown. The precise distance at which an infected individual can transmit infectious SARS-CoV-2 is also unknown. However, it would be reasonable to assume that the risk of transmission is reduced the farther one is away from the infected individual [60]. The extent to which the severity of infection influences the risk of transmission is uncertain.

### Towards standardized methodologies for airborne viral culture studies

The heterogeneity of sampling approaches, culture techniques, and reporting practices in the existing literature underscores the need for standardized methods. Based on established aerobiological principles and prior experimental work, we propose several core components for a standardized protocol.

1. **Sampler selection is essential.** Studies aiming to assess infectivity should prioritize liquid impingers or other liquid-based collectors. As demonstrated in aerobiology literature, including the comprehensive review by Verreault and colleagues [56], filters, while efficient at capturing particles, often cause desiccation and structural damage to enveloped viruses, substantially reducing culturability. Liquid-based systems preserve viral structure and maintain infectivity by capturing aerosolized particles directly into buffered media.
2. **Environmental conditions must be recorded rigorously.** Relative humidity (RH) and temperature strongly influence viral stability in aerosols. For SARS-CoV-2 and other enveloped viruses, RH-dependent decay is well documented. Without these data, negative culture results cannot be interpreted as environmental inactivation versus the true absence of viable virus.
3. **Sampling systems should be validated prior to field deployment.** Validation using enveloped-virus surrogates, such as bacteriophage Phi6, enables quantification of recovery efficiency and distinguishes methodological limitations from genuine environmental findings.
4. **Reporting standards** should include predefined sampling distances and heights, standardized air volumes and sampling durations, documentation of storage and transport conditions, and calibrated RT-PCR results expressed as RNA copies per cubic meter. When feasible, viral culture should be complemented by genomic sequencing to confirm identity and source linkage. The viral cultures from samples should also be appropriately quantified.

Together, these elements provide a practical and feasible framework for future airborne viral infectivity studies and support the development of international, multidisciplinary guidelines.

## Conclusions

The proportion of positive SARS-CoV-2 viral cultures following positive RNA samples in the air is low, suggesting that while viral RNA may be present, the likelihood of detecting culturable, infectious viruses is substantially lower. Paucity of data prevents us from drawing conclusions about the threshold for viral infectiousness, as well as the relationship between distance and infectiousness. Discrepancies in the metrics used to determine viral load make comparison of research results across studies challenging. Therefore, the development of standardized guidelines for sampling and culturing viral particles from the air is warranted. To build a more robust evidence base, future studies investigating airborne transmission of viruses should consistently perform viral culture and genome sequencing to confirm infectivity and transmission chains.

## Electronic supplementary material

Below is the link to the electronic supplementary material.

Supplementary Material 1

Supplementary Material 2

Supplementary Material 3

Supplementary Material 4

Supplementary Material 5

## Data Availability

All data generated or analysed during this study are included in this published article [and its supplementary information files].
