# Supplementary material for: Viral cultures for assessing airborne infectiousness of SARS-CoV-2: a systematic review and meta-analysis
Source: BMC Infect Dis. 2025 Dec 25;26:297. doi: 10.1186/s12879-025-12430-z (PMC12888525; doi:10.1186/s12879-025-12430-z)
Supplement: Supplementary file 1 — Supplementary Material 1 [file 12879_2025_12430_MOESM1_ESM.docx]

**Appendix 1: Search Strategies**

| **Initial Search** |
| --- |
| **WHO** |
| Search: (fomite OR surfaces OR orofecal OR oro-fecal OR airbourne OR inhalation OR airborne OR air OR faecal OR aerosol OR fecal OR vertical OR blood OR droplet OR "viral load" OR "viral threshold") AND transmission |
| last article: Congenital and Intrapartum SARS-CoV-2 Infection in Neonates: Hypotheses, Evidence and Perspectives. |
|  |
| **LitCovid** |
| (fomite OR surfaces OR orofecal OR oro-fecal OR inhalation OR airborne OR air OR faecal OR aerosol OR fecal OR vertical OR blood OR droplet OR "viral load" OR "viral threshold") AND transmission |
|  |
| **Scholar** |
| (fomite OR surfaces OR orofecal OR oro-fecal OR inhalation OR airborne OR air OR faecal OR aerosol OR fecal OR vertical OR blood OR droplet OR "viral load" OR "viral threshold") AND transmission AND (coronavirus OR covid-19 OR SARS-CoV-2) |
|  |
| **medRxiv** |
| site:medrxiv.org (fomite OR surfaces OR orofecal OR oro-fecal OR inhalation OR airborne OR air OR faecal OR aerosol OR fecal OR vertical OR blood OR droplet OR "viral load" OR "viral threshold") AND transmission AND (coronavirus OR covid-19 OR SARS-CoV-2) |
|  |
| **Revised search (just airborne)** |
|  |
| **WHO**: no longer available from 2024 |
|  |
| **LitCovid** |
| (inhalation OR airborne OR air OR aerosol OR droplet) AND ("viral load" OR "viral threshold" OR "viral culture" OR "virus culture" OR vero) AND transmission |
|  |
| **Scholar** |
| (inhalation OR airborne OR air OR aerosol OR droplet) AND ("viral load" OR "viral threshold" OR "viral culture" OR "virus culture" OR vero) AND transmission AND (coronavirus OR covid-19 OR SARS-CoV-2) |
| Scholar done to page 22 of results (3 pages with no good hits) |
|  |
| **medRxiv** |
| site:medrxiv.org (inhalation OR airborne OR air OR aerosol OR droplet) AND ("viral load" OR "viral threshold" OR "viral culture" OR "virus culture" OR vero) AND transmission AND (coronavirus OR covid-19 OR SARS-CoV-2) |
