# Supplementary material for: Viral cultures for assessing airborne infectiousness of SARS-CoV-2: a systematic review and meta-analysis
Source: BMC Infect Dis. 2025 Dec 25;26:297. doi: 10.1186/s12879-025-12430-z (PMC12888525; doi:10.1186/s12879-025-12430-z)
Supplement: Supplementary file 2 — Supplementary Material 2 [file 12879_2025_12430_MOESM2_ESM.docx]

**Appendix 2: Details of excluded studies including the reasons for exclusion**

| **Study ID** | **Title** | **Weblink** | **DOI** | **Reason for exclusion** |
| --- | --- | --- | --- | --- |
| Comber L 2020 | Airborne transmission of SARS-CoV-2 via aerosols | <https://onlinelibrary.wiley.com/doi/10.1002/rmv.2184> | 10.1002/rmv.2184 | Review |
| Dohla M 2022 | SARS-CoV-2 in Environmental Samples of Quarantined Households | <https://www.mdpi.com/1999-4915/14/5/1075> | 10.3390/v14051075 | No culture of air samples |
| Dumont-Leblond N 2021 | Positive no-touch surfaces and undetectable SARS-CoV-2 aerosols in long-term care facilities: An attempt to understand the contributing factors and the importance of timing in air sampling campaigns | <https://www.ajicjournal.org/article/S0196-6553(21)00059-6/fulltext> | 10.1016/j.ajic.2021.02.004 | No culture of air samples |
| Fortin A 2022 | Detection of infectious SARS-CoV-2 in frozen aerosol samples collected from hospital rooms of patients with COVID-19 | <https://www.medrxiv.org/content/10.1101/2022.11.14.22282295v1> | 10.1101/2022.11.14.22282295 | Pre-print of included study |
| Qutub M 2022 | Duration of viable SARS-CoV-2 shedding from respiratory tract in different human hosts and its impact on isolation discontinuation polices revision; a narrative review | <https://pmc.ncbi.nlm.nih.gov/articles/PMC8848577/> | 10.1016/j.clinpr.2022.100140 | Review |
| Gomes da Silva P 2021 | Airborne spread of infectious SARS-CoV-2: Moving forward using lessons from SARS-CoV and MERS-CoV | <https://www.sciencedirect.com/science/article/pii/S0048969720363312> | 10.1016/j.scitotenv.2020.142802 | Review |
| Borges JT 2021 | SARS-CoV-2: a systematic review of indoor air sampling for virus detection | <https://link.springer.com/article/10.1007/s11356-021-13001-w> | 10.1007/s11356-021-13001-w | Review |
| Cherrie JW 2021 | Contamination of Air and Surfaces in Workplaces with SARS-CoV-2 Virus: A Systematic Review | <https://academic.oup.com/annweh/article/65/8/879/6331478?login=false> | 10.1093/annweh/wxab026 | Review |
| Dabisch PA 2022 | Comparison of the survival of different isolates of SARS-CoV-2 in evaporating aerosols | <https://www.tandfonline.com/doi/full/10.1080/02786826.2022.2128712> | 10.1080/02786826.2022.2128712 | Laboratory experiment |
| Gomes da Silva P 2023 | Airborne SARS-CoV-2 is more frequently detected in environments related to children and elderly but likely non-infectious, Norway, 2022 | <https://virologyj.biomedcentral.com/articles/10.1186/s12985-023-02243-4> | 10.1186/s12985-023-02243-4 | No culture of air samples |
| Ratnesar-Shumate S 2021 | Comparison of the performance of aerosol sampling devices for measuring infectious SARS-CoV-2 aerosols | <https://www.tandfonline.com/doi/full/10.1080/02786826.2021.1910137> | 10.1080/02786826.2021.1910137 | Wrong study design |
| Alsved M 2023 | Infectivity of exhaled SARS-CoV-2 aerosols is sufficient to transmit covid-19 within minutes | <https://www.nature.com/articles/s41598-023-47829-8> | 10.1038/s41598-023-47829-8 | Wrong study design |
| Barbieri P 2021 | Molecular detection of SARS-CoV-2 from indoor air samples in environmental monitoring needs adequate temporal coverage and infectivity assessment | <https://www.sciencedirect.com/science/article/pii/S0013935121004941?via%3Dihub> | 10.1016/j.envres.2021.111200 | Wrong study design |
| Fox-Lewis A 2024 | Chapter 11 - Airborne transmission of SARS-CoV-2 | <https://www.sciencedirect.com/science/article/abs/pii/B9780323956468000500> | 10.1016/B978-0-323-95646-8.00050-0 | Review |
| Derqui N 2023 | Risk factors and vectors for SARS-CoV-2 household transmission: a prospective, longitudinal cohort study | <https://www.thelancet.com/journals/lanmic/article/PIIS2666-5247(23)00069-1/fulltext> | 10.1016/S2666-5247(23)00069-1 | Did not examine air samples |
| Oswin HP 2022 | The dynamics of SARS-CoV-2 infectivity with changes in aerosol microenvironment | <https://www.pnas.org/doi/full/10.1073/pnas.2200109119> | 10.1073/pnas.2200109119 | Review |
| Adzic F 2022 | A post-occupancy study of ventilation effectiveness from high-resolution CO(2) monitoring at live theatre events to mitigate airborne transmission of SARS-CoV-2 | <https://www.sciencedirect.com/science/article/pii/S0360132322006254?via%3Dihub> | 10.1016/j.buildenv.2022.109392 | No culture of air samples |
| Azizi Jalilian F 2022 | Evaluation of SARS-CoV-2 in Indoor Air of Sina and Shahid Beheshti Hospitals and Patients' Houses | <https://link.springer.com/article/10.1007/s12560-022-09515-2> | 10.1007/s12560-022-09515-2 | No culture of air samples |
| Coleman KK 2021 | Viral Load of Severe Acute Respiratory Syndrome Coronavirus 2 (SARS-CoV-2) in Respiratory Aerosols Emitted by Patients With Coronavirus Disease 2019 (COVID-19) While Breathing, Talking, and Singing | <https://academic.oup.com/cid/article/74/10/1722/6343417?login=false> | 10.1093/cid/ciab691 | No culture of air samples |
| Cordery R 2022 | Transmission of SARS-CoV-2 by children to contacts in schools and households: a prospective cohort and environmental sampling study in London | <https://www.thelancet.com/journals/lanmic/article/PIIS2666-5247(22)00124-0/fulltext> | 10.1016/S2666-5247(22)00124-0 | No culture of air samples |
| Correia G 2022 | SARS-CoV-2 air and surface contamination in residential settings | <https://www.nature.com/articles/s41598-022-22679-y> | 10.1038/s41598-022-22679-y | No culture of air samples |
| Vosoughi M 2021 | Investigation of SARS-CoV-2 in hospital indoor air of COVID-19 patients’ ward with impinger method | <https://link.springer.com/article/10.1007/s11356-021-14260-3#citeas> | 10.1007/s11356-021-14260-3 | No culture of air samples |
| Krambrich J 2021 | SARS-CoV-2 in hospital indoor environments is predominantly non-infectious | <https://virologyj.biomedcentral.com/articles/10.1186/s12985-021-01556-6> | 10.1186/s12985-021-01556-6 | No culture of air samples |
| Li H 2022 | Environmental Surveillance for SARS-CoV-2 in Two Restaurants from a Mid-scale City that Followed U.S. CDC Reopening Guidance | <https://aaqr.org/articles/aaqr-21-11-covid2-0304> | 10.4209/aaqr.210304 | No culture of air samples |
| Navas M-C 2023 | Outbreak report of SARS-CoV-2 infection by airborne transmission: Epidemiologic and molecular evidence | <https://revistabiomedica.org/index.php/biomedica/article/view/6695/5316> | 10.7705/biomedica.6695 | Did not examine air samples |
| Puhach O 2022 | Infectious viral load in unvaccinated and vaccinated individuals infected with ancestral, Delta or Omicron SARS-CoV-2 | <https://www.nature.com/articles/s41591-022-01816-0> | 10.1038/s41591-022-01816-0 | Did not examine air samples |
| Zupin L 2021 | Evaluation of Residual Infectivity after SARS-CoV-2 Aerosol Transmission in a Controlled Laboratory Setting | <https://www.mdpi.com/1660-4601/18/21/11172> | 10.3390/ijerph182111172 | Laboratory experiment |
| Adenaiye OO 2021 | Infectious Severe Acute Respiratory Syndrome Coronavirus 2 (SARS-CoV-2) in Exhaled Aerosols and Efficacy of Masks During Early Mild Infection | <https://academic.oup.com/cid/article/75/1/e241/6370149?login=false> | 10.1093/cid/ciab797 | Exhaled breath samples |
| de Sousa NR 2024 | Detection of active SARS-CoV-2 in cough aerosols from COVID-19 patients | <https://www.tandfonline.com/doi/full/10.1080/23744235.2024.2374307#abstract> | 10.1080/23744235.2024.2374307 | Cough samples |
| Gallichotte EN 2022 | Detection of SARS-CoV-2 in exhaled air using non-invasive embedded strips in masks | <https://pmc.ncbi.nlm.nih.gov/articles/PMC9329094/> | 10.1016/j.ajic.2022.01.010 | Exhaled breath samples |
| Jaumdally S 2024 | Frequency, kinetics and determinants of viable SARS-CoV-2 in bioaerosols from ambulatory COVID-19 patients infected with the Beta, Delta or Omicron variants | <https://www.nature.com/articles/s41467-024-45400-1> | 10.1038/s41467-024-45400-1 | Cough samples |
| Johnson TJ 2022 | Viral load of SARS-CoV-2 in droplets and bioaerosols directly captured during breathing, speaking and coughing | <https://www.nature.com/articles/s41598-022-07301-5> | 10.1038/s41598-022-07301-5 | Breathing, speaking and coughing samples |
| Lin Y-C 2022 | Detection and quantification of infectious severe acute respiratory coronavirus-2 in diverse clinical and environmental samples | <https://www.nature.com/articles/s41598-022-09218-5> | 10.1038/s41598-022-09218-5 | Cough samples |
| Tham KW 2022 | Differential aerosol shedding of SARS-CoV-2 Delta and Omicron variants during respiratory activities | <https://europepmc.org/article/ppr/ppr452953> | 10.21203/rs.3.rs-1348915/v1 | Exhaled breath samples |
| Lai J 2023 | Exhaled Breath Aerosol Shedding of Highly Transmissible Versus Prior Severe Acute Respiratory Syndrome Coronavirus 2 Variants | <https://academic.oup.com/cid/article/76/5/786/6773834?login=false> | 10.1093/cid/ciac846 | Exhaled breath samples |
| Tan KS 2023 | SARS-CoV-2 Omicron variant shedding during respiratory activities | <https://www.ijidonline.com/article/S1201-9712(23)00109-1/fulltext> | 10.1016/j.ijid.2023.03.029 | Exhaled breath |
