# Supplementary material for: Viral cultures for assessing airborne infectiousness of SARS-CoV-2: a systematic review and meta-analysis
Source: BMC Infect Dis. 2025 Dec 25;26:297. doi: 10.1186/s12879-025-12430-z (PMC12888525; doi:10.1186/s12879-025-12430-z)
Supplement: Supplementary file 3 — Supplementary Material 3 [file 12879_2025_12430_MOESM3_ESM.pptx]

## Slide 1
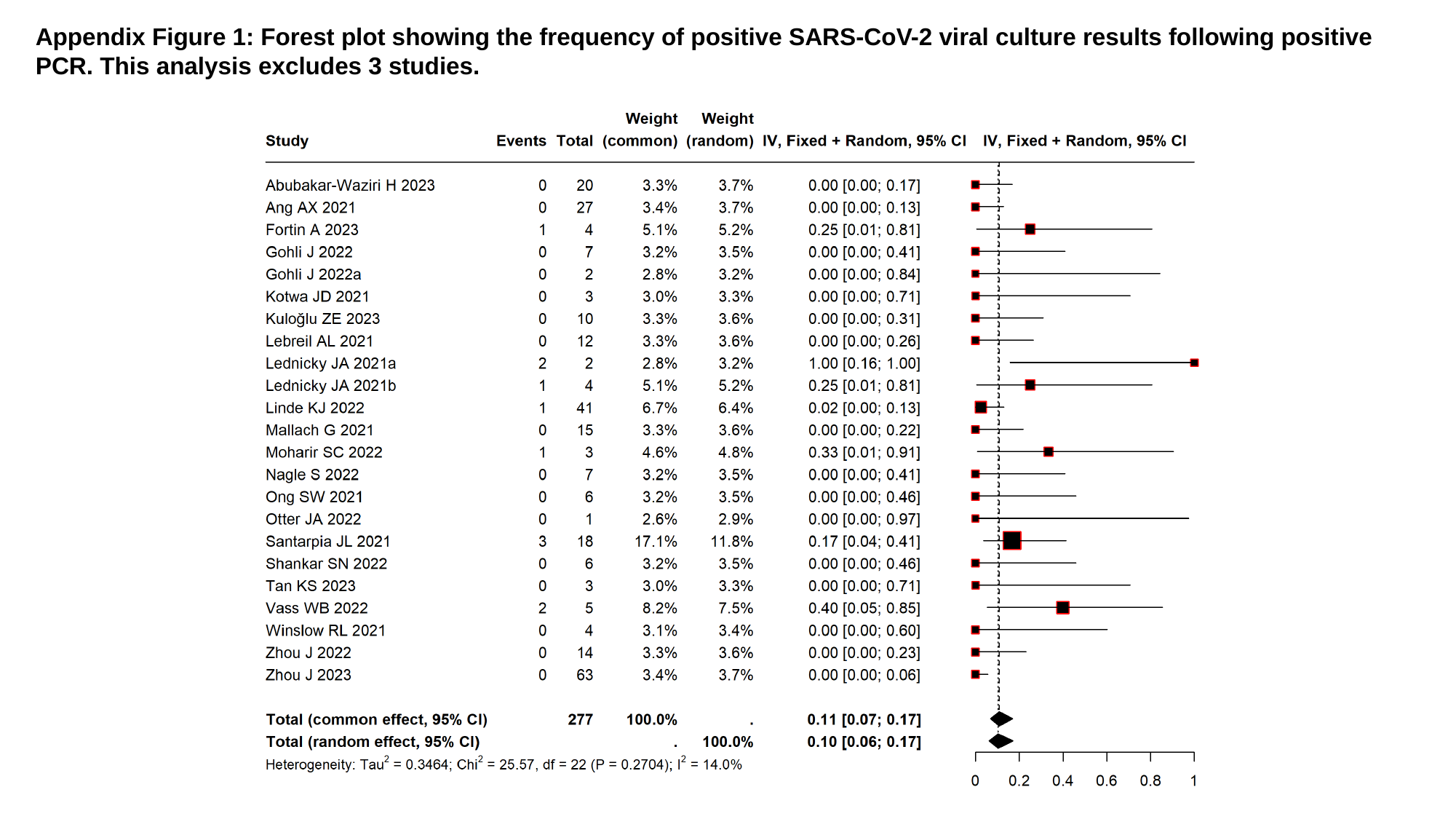

Appendix Figure 1: Forest plot showing the frequency of positive SARS-CoV-2 viral culture results following positive PCR. This analysis excludes 3 studies.
