# Supplementary material for: Viral cultures for assessing airborne infectiousness of SARS-CoV-2: a systematic review and meta-analysis
Source: BMC Infect Dis. 2025 Dec 25;26:297. doi: 10.1186/s12879-025-12430-z (PMC12888525; doi:10.1186/s12879-025-12430-z)
Supplement: Supplementary file 4 — Supplementary Material 4 [file 12879_2025_12430_MOESM4_ESM.pptx]

## Slide 1
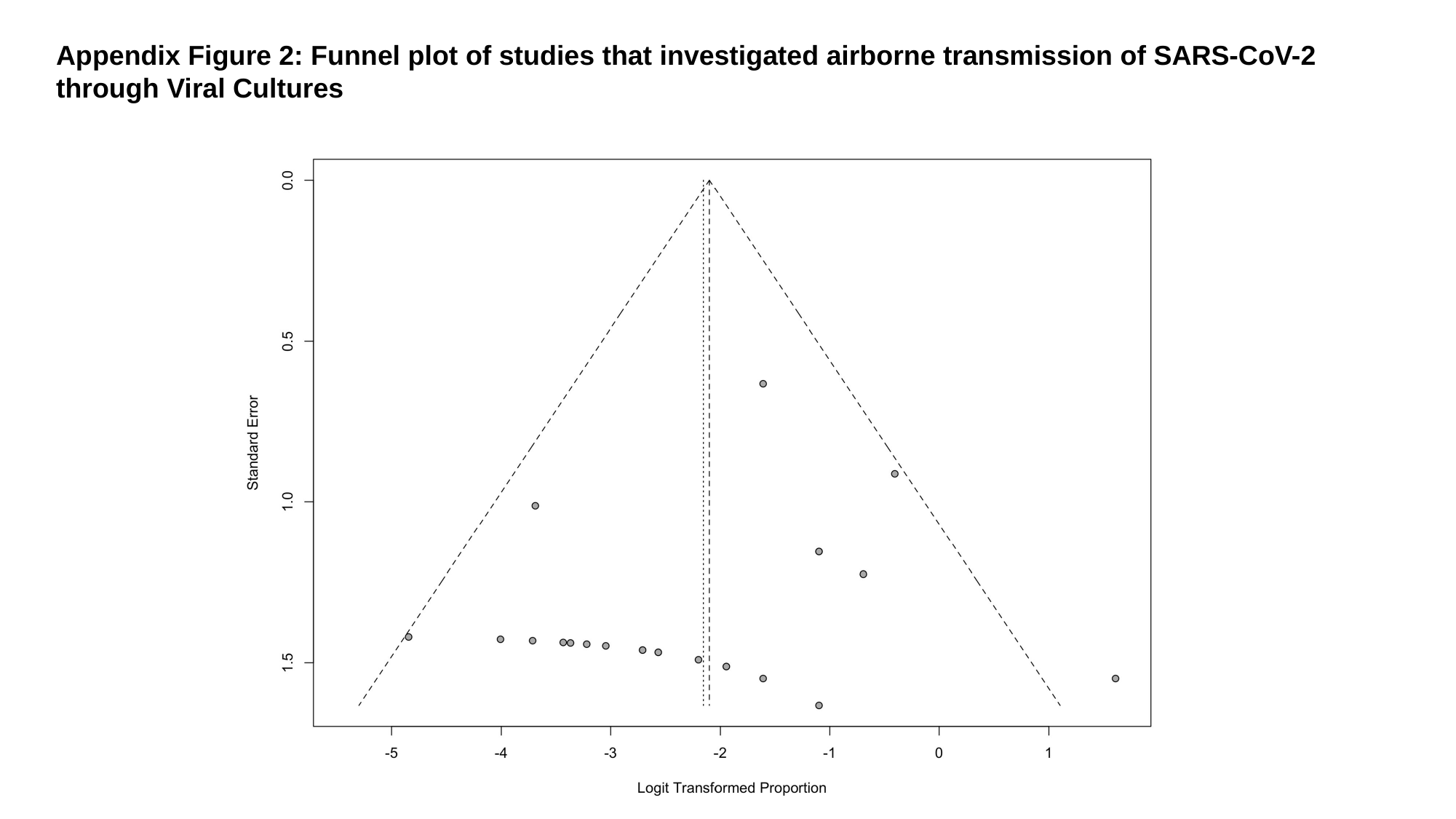

Appendix Figure 2: Funnel plot of studies that investigated airborne transmission of SARS-CoV-2 through Viral Cultures
