# Supplementary material for: Viral cultures for assessing airborne infectiousness of SARS-CoV-2: a systematic review and meta-analysis
Source: BMC Infect Dis. 2025 Dec 25;26:297. doi: 10.1186/s12879-025-12430-z (PMC12888525; doi:10.1186/s12879-025-12430-z)
Supplement: Supplementary file 5 — Supplementary Material 5 [file 12879_2025_12430_MOESM5_ESM.docx]

**Appendix Table 1: Additional details of studies that assessed airborne transmission of SARS-CoV-2 through viral cultures**

| **Study ID** | **Setting** | **Source population (no. of people), CT and symptomatic or asymptomatic** | **Air Sampling methods** | **Sampling distance** | **Duration of sampling** | **Proportion of positive cultures (%)** | **Results of viral quantification** | **Results of sequencing** |
| --- | --- | --- | --- | --- | --- | --- | --- | --- |
| Abubakar-Waziri H 2023 [8] | Hospital | ICUs with adults suffering from COVID-19, respiratory wards, and public waiting areas. | Liquid-based total suspended particulate (TSP) samplers, size-segregated particulate matter (PM) samplers and portable samplers. | Not reported | 8 months: Between 10 March 2021 and 14 December 2021 | 0 | Ranged from 118 virus copies/m^3^ of air to 707,284 copies/m^3^ across all samples | 2 samples from CDU underwent successful genomic sequencing |
| Ang AX 2021 [9] | Hospital | 21 COVID-19 patients in naturally ventilated open cohort wards (2) and mechanically ventilated isolation ward (1), All patients were mildly symptomatic | Filter-based SASS 3100 air samplers | 0.9-3m from patients | February to May 2020 - during the peak of the first wave | 0 | NR | NR |
| de Sousa NR 2022 [10] | Hospital | Infectious disease wards: 15 patients with confirmed COVID-19. One patient was asymptomatic; the remaining patients were symptomatic | Tuberculosis Hotspot detector (THOR) electrostatic air sampler | ≥2m from patients | Four separate occasions (December 10, 2020, January 19, 2021, January 21, 2021, and February 23, 2021) | 0.56 | SARS-CoV−2+ PFUs/ml (IQR, SD): 40 (40–40; 26.7) in patient’s room; 80 (40–120; 37.7) in anteroom | NR |
| Fortin A 2023 [11] | Hospital | Patients with acute COVID-19. Samples were stored for 14 months before culture. | Two types of samplers, namely 37mm closed-face cassettes with 0.8μm polycarbonate filters (SKC, Eighty-Four) or a condensation growth tube (CGT) air sampler (Series 110A Liquid Spot Sampler, Aerosol Devices) | 2-3m from the patient's bed | Fall of 2020 | 0.25 | The titer of virions in the supernatant produced by sample #4 after 2 cycles of infection was 6.32 x 10^7^ TCID50/mL | NR |
| Gohli J 2022 [12] | Testing room | 14 subjects that had recently tested positive for SARS-CoV-2. None to mild/moderate respiratory symptoms. Ct was 32-38 for COVID-positive. | SASS 3100 air samplers. Air sampling was conducted on average 6 days (median = 12; range 2–15) after symptom onset; no data for 3/14 subjects | Two in parallel at 1m distance, two in parallel at 2m distance, and a single air sampler at 4m distance. | October 14th, December 6th, 2020, January 17th, and March 14, 2021 | 0 | NR | N/A |
| Gohli J 2022a [13] | University hospital | 13 COVID-19 patients in infectious disease ward, all symptomatic and on nebulizer therapy. Ct was 32 and 35 for COVID-positive in 2 patients | SASS 3100 air samplers. Air sampling was conducted on average 13.4 days (median = 12; range 7–23) after symptom onset. Air samples from the isolation rooms were collected immediately prior to and during NT. | 1.2m height from foot of patient's bed | November 2020 to January 2021 | 0 | NR | N/A |
| Kitagawa H 2022 [14] | Hospital | 20 patients with PCR-confirmed COVID-19. All patients have had COVID-19 symptoms for ≤7 days | MD8 Airport. The windows of the room were closed during the air sampling | 1m from patient's head | July 20 to October 31, 2021 | 0.28 | Median concentration: 8.9 × 10^2^ TCID50/m^3^. The RNA concentration was significantly higher in samples in which viable SARS-CoV-2 was detected vs. samples in which there was no viable virus (P=0.027) | WGS performed on 12 of 18 SARS-CoV-2 strains isolated from NPS samples, which confirmed that all strains belonged to the B.1.617.2 lineage (Delta variant). |
| Kotwa JD 2021 [15] | Hospital | 78 inpatients with COVID-19 at 6 acute care hospitals, all were symptomatic. median Ct value among positive follow-up NP swabs was 31.4. | Air sampling pump (GilAir Plus Personal Air Sampling Pump, Sensidyne, St. Petersburg, FA). The median time between onset of illness and air sampling was 11 days (IQR, 7–14); the time between onset of illness and sampling date for all 3 PCR-positive air samples was 4 days. | 1m and 2m from patient's head | Between March and May 2020 | 0 | NR | Not performed due to poor quality sequences |
| Kuloğlu ZE 2023 [16] | University hospital | 16 participants, three of whom tested positive for the Omicron variant on the same day. | Coriolis µ-biological air sampler. The aerosol samples in the meeting room were collected 1 hour, 2.5 hours, 4.5 hours, and 24 hours after the meeting. | For hospital patients, 2m away from the patients in COVID-19 and ICU wards. | 3rd of January 2022 and the 25th of March 2022 | 0 | 1.80 × 10^2^ PFU m–3 and 2.80 × 108 PFU m–3 in patient's area; 1.45 × 102 PFU m–3 and 3.6 × 1010 PFU m–3 in patients' rooms. Significant difference between the mean viral loads in rooms of patients wearing masks vs. no masks: (1.45 × 10^2^ PFU m–3 vs. 3.60 × 10^10^ PFU m–3 | NR |
| Lebreil AL 2021 [17] | Hospital | 20 COVID-19 patients in ICU rooms. All had COVID-19–induced ARDS and required an oxygen supplementation delivered by an HFNC or by AMV | Sartorius MD8 air sampler MD8 Airscan. Samples were collected at 0800, corresponding to a delay of 15 hours after the last routine surface cleaning and disinfection | Not reported | 8 April to 30 June 2020 | 0 | No viral RNA load increase was observed in the supernatants of cell culture 4 and 9 days after inoculation of air samples | NR |
| Lednicky JA 2021a [18] | Clinic in University Hospital | Patients suspicious for COVID-19 with symptoms, travel history or known exposure to COVID-19 positive patients | The VIVAS. | 3m from nearest patient traffic | March 20, 2020 | 1 | Estimated SARS-CoV-2 concentration was 0.87 virus genomes L^–1^ air. | The complete SAR-CoV-2 genomic sequence most closely resembled SARS-CoV-2 genomes previously described. Collection of a positive sample from a distance more than 2 m away from the nearest patient traffic implies the virus was in an aerosol. |
| Lednicky JA 2021b [19] | Car | 1 COVID-19 patient with mild illness | Sioutas Personal Cascade impactor sampler clipped onto the sun-visor above the passenger seat next to patient | Approx. 0.9m from the patient’s face | 15 minutes (drive from the clinic to home) | 0.25 | Amount of virus present in 390 L of sampled air was approx. 340 virus genome equivalents. Estimated SARS-CoV-2 concentration was 0.87 virus genomes L^–1^ air | The virus isolated was designated as SARS-CoV-2/human/USA/UF-29/2020 |
| Linde KJ 2022 [20] | Nursing home | 13 COVID-19 patients | Conical Inhalable dust Sampler (CIS). Patients included within 8 days since the onset of symptoms or within 8 days since the first positive surveillance test result. | 0.5-2m away from patients | December to April | 0.02 | NR | >90% of the reference was covered and uploaded in GISAID for OPS. All variants were B.1.221, a known variant, circulating in The Netherlands at the time of the study. Samples collected at the same location were closely genetically related. |
| Mallach G 2021 [21] | Hospitals and long-term care homes | COVID-19 patients (confirmed by qRT-PCR) in 99 rooms located in ICUs, hospital ward rooms, rooms in long-term care facilities and at a correctional institute. | Ultrasonic Personal Air Samplers | Between 2 and 3m from the patient | Between September 22, 2020 and January 25, 2021 | 0 | The mean RNA concentration in the air was 1202.4 copy numbers/m^3^ (range 63.8–11939.9; SE 977.2) |  |
| Moharir SC 2022 [22] | Hospitals and community | COVID-19 patients who were either asymptomatic or mildly symptomatic. Ct of positive NP swabs was 19.85 to 31.76. | AirPort MD8 air sample, or Merck's MAS-100® series air sampler | 1.2-3.7m from COVID-19 patients | Between September 2020 to January 2021, between July 2020 to December 2020 | 0.33 | NR | NR |
| Nagle S 2022 [23] | Hospital | 65 COVID-19 patients (confirmed by qRT-PCR), all symptomatic. Median Ct of NP swabs was 19. | Coriolis µ air sampler | 1m and 3m from the patient's head | 22nd January to 8th April 2021 | 0 | NR | NR |
| Ong SW 2021 [24] | Hospital rooms and community isolation facilities | 20 COVID-19 patients - 12 (63.2%) patients were symptomatic on the day of sampling. | BioSpot-VIVAS BSS300-P bioaerosol sampler. The median day of illness was day 5 (IQR, 4–7) | 1m from the patient's bed. | Not reported | 0 | Across positive air samples, concentrations of virus copies/m^3^ of air ranged from 178.9 to 2,738.4 | Virus cultures of all 7 qRT-PCR–positive BioSpot air samples were negative after 4 blind passages. |
| Otter JA 2022 [25] | Acute healthcare setting | ED, ICU, acute admissions unit, ward, entrance and public area | Coriolis Micro air sampler | Not reported | Apr-20 | 0 | NR | NR |
| Santarpia JL 2021 [26] | Hospital | 6 COVID-19 patients | BC251 aerosol sampler | Foot of each patient’s bed | Three separate days in April of 2020 | 0.17 | Cultures inoculated with known virus showed statistically significant replication at 10^−1^ pfu/mL and 10^-0^ PFU/mL | NR |
| Shankar SN 2022 [27] | Residential rooms of self-isolating persons | Two 20-year-old persons with symptomatic COVID-19 | NIOSH bioaerosol sampler (BC-251), and the VIVAS. Sampling was between 2 and 5 days of symptom onset | 0.3–2.2m | September 22 to 25, 2020 | 0 | Viral concentrations were estimated to be 1.19E+05 GE/cm^3^ to 3.04E+05 GE/cm^3^ of air | Unclear |
| Tan KS 2023 [28] | Hospital | Patients with COVID-19 (confirmed by qRT-PCR). All patients were admitted after a positive COVID-19 test by RT-qPCR (with cycle threshold, Ct of < 25 | AerosolSenseTM air sampling device. | <2m, <2.5m, <10m, or <14m away from the patient | January to March 2022 | 0 | ≥40 RNA copies/m3 of air for positive RNA samples | NR |
| Vass WB 2022 [29] | Residential room of self-isolating person | 1 symptomatic COVID-19 patient | A BioSpot Viable Virus Aerosol Sampler and two NIOSH bioaerosol samplers. Sample collection on day 3 following onset of symptoms | 2.5m or 3m from centre of bed headboard | September 3, 2021 | 0.4 | The concentrations of viable viruses were 132 and 292 PFU/L of air | A complete consensus SARS-CoV-2 genomic sequence was obtained for all virus isolates, and the sequences were identical. |
| Vass WB 2023 [30] | Residential settings of individuals with COVID-19 | Seven volunteers, of which one resided in a single-family home and the others in multi-story complexes | BioSpot-VIVAS, VIVAS, and BC-251. | 1.5–1.8m from patients | January to May 2022 | 0.33 | Not specified | Sampler type could not be included as a variable in the viable virus model |
| Winslow RL 2021 [31] | Hospital | 30 hospitalised patients with COVID-19 requiring supplemental oxygen. Ct for positive NP samples was <45 | Coriolis micro air sampler | 50 cm from mouth of patient | Between 11 December 2020 and 19 February 2021 | 0 | Not specified | N/A |
| Zhou J 2022 [32] | Acute healthcare setting | Adult patients with COVID-19 in seven clinical areas (ED, an admissions ward, 2 COVID-19 cohort wards, theatres during tracheostomy procedures, an admissions ward, (ICU), and a 6-bed bay converted into a negative-pressure area for management of CPAP on patients with COVID-19) and a public area of the hospital | Coriolis μ air sampler | Not reported | Between 2 April 2020 and 20 April 2020 | 0 | Not performed | NR |
| Zhou J 2023 [33] | Hospital | 36 participants innoculated with 10 50% tissue culture infectious dose (TCID50) of pre-alpha wild-type SARS-CoV-2 (Asp614Gly virus isolated in 2020) by nasal drops. Ct ≤35 was used as the threshold for positive NP swab test. | Coriolis μ air sampler | Approx. 1m from patient's head | Between March 6 and July 8, 2021 | 0 | NR | NR |
